# TREM2 in Urological Malignancies and Benign Lesions: Mechanistic Convergence, Functional Heterogeneity, and Translational Perspectives: A Narrative Review

**DOI:** 10.3390/cancers18030359

**Published:** 2026-01-23

**Authors:** Yu Dai, Yaqiang Feng, Cheng Wang, Helin Zhang, Panfeng Shang

**Affiliations:** 1 Department of Urology, Lanzhou University Second Hospital, 82 Cuiyingmen, Chengguan District, Lanzhou 730030, China; 2 Department of Urology, Baoji People’s Hospital, Baoji 721000, China

**Keywords:** TREM2, urological neoplasms, benign prostatic hyperplasia, PI3K/AKT pathway, tumor microenvironment, immunotherapy

## Abstract

Triggering receptor expressed on myeloid cells-2 (TREM2) is a key myeloid immune checkpoint for macrophage plasticity. However, its functional landscape in urology is still incomplete. This review addresses this gap by providing the first systematic synthesis of TREM2 in urological malignancies (bladder, prostate, and renal cell carcinomas) and benign conditions. We find a strong correlation between TREM2 upregulation and adverse clinical outcomes in these cancers. Importantly, we highlight the phenomenon of “mechanistic convergence”: unlike the high context-dependency of other organ systems, TREM2 appears to drive progression in urological malignancies by a common convergent signaling hub, the PI3K/AKT pathway. This contrasts sharply with its metabolic role in benign prostatic hyperplasia and its protective role in non-malignant renal injury. We also consider the translational potential of TREM2 as a prognostic biomarker (specifically urine detection) and as a therapeutic target to reverse immunotherapy resistance.

## Simple Summary

Urinary system cancers, including bladder, prostate, and kidney cancers, impose a substantial global health burden. Although immunotherapy benefits certain patients, many ultimately develop resistance to these drugs. This review examines the role of the TREM2 protein in these diseases. TREM2 functions as a double-edged sword. In urinary system cancers, it generally promotes tumor growth and suppresses anti-tumor immunity through a common mechanism involving the PI3K/AKT pathway. Conversely, in cases of benign kidney injury, it may exert a protective effect. Additionally, TREM2 is detectable in urine, presenting a potential noninvasive method for disease surveillance. Clarifying this dual role could facilitate the development of combined strategies involving immunotherapy and targeted drugs, ultimately improving patient survival.

## 1. Introduction

Bladder cancer, prostate cancer, and renal cell carcinoma are among the most lethal malignancies of the urinary system globally. Although the advent of immunocheckpoint inhibitors (ICI) revolutionized the treatment of some patients, primary or acquired resistance remains a challenging clinical problem [1,2]. Several studies show that myeloid cell immunosuppression in the TME is an important factor in immune evasion and failure [3]. Among various myeloid immunomodulatory molecules, triggering receptor expressed on myeloid cells-2 (TREM2) has been gaining attention recently.

TREM2, a member of the immunoglobulin superfamily primarily expressed on myeloid cells such as macrophages, transduces intracellular signals via the adaptor protein DAP12, thereby exerting core functions in immune regulation, lipid metabolism, and tissue homeostasis [4,5]. In the oncological context, TREM2 is highly expressed on tumor-associated macrophages (TAMs) and has been substantiated in various solid tumors—including lung and ovarian cancer—as a crucial determinant in shaping an immunosuppressive TME, promoting progression and correlating with poor patient outcomes [6,7]. However, TREM2 function has a high context dependence; it may exert protective effects in Alzheimer’s disease or some other malignancies, suggesting its complexity in different pathophysiologies [8]. Although research in urological oncology is growing in importance, studies regarding TREM2 in this field remain fragmented and not integrated. In addition, the new role of TREM2 in benign urological lesions (BPH) offers a unique opportunity to study its function. To our knowledge, this review is the first systematic review explicitly proposing the notion of “mechanistic convergence” of TREM2 in Urology. Unlike previous work on TREM2 in cancer and neurodegeneration [9,10], we compare the consistent tumorigenic role of TREM2 in urological malignancies with distinct immunometabolic functions in benign tumors. By highlighting the PI3K/AKT pathway as a central convergence target, we provide a framework for understanding the “double-edged” nature of TREM2 in the urinary system. In this review we describe the biochemical features of TREM2; summarize its expression pattern, clinical significance, and functional pathways in bladder, prostate, and renal cancers; discuss its emerging role in benign urological disease; and assess the translational prospects of TREM2 as a biomarker and therapeutic target.

### Literature Search Methodology

To summarize, we searched for relevant studies in PubMed and Google Scholar up to December 2025 using Medical Subject Headings and free text synonyms based on standard terms. The search strategy employed a combination of Medical Subject Headings (MeSH) and free-text synonyms derived from standard terminology. Key search terms included “TREM2” combined with urological malignancy-specific terms such as “Prostatic Neoplasms” (e.g., prostate cancer, prostatic cancer), “Urinary Bladder Neoplasms” (e.g., bladder cancer, transitional cell carcinoma), and “Kidney Neoplasms” (e.g., renal cell carcinoma, Wilms tumor) and benign conditions like “Benign Prostatic Hyperplasia” and “Acute Kidney Injury”. The search included foundational studies as well as experimental and clinical studies published in the last five years. Non-English publications and abstracts without full text were excluded.

## 2. Biological Functions and Signaling Pathways of TREM2

TREM2 is a type I transmembrane glycoprotein encoded on human chromosome 6, with five exons and around 230 amino acids. It consists of an extracellular V-type immunoglobulin domain, a single transmembrane region, and a short tail with no signaling motifs. It recognizes ligands like lipids and apolipoproteins, whereas intracellular signaling is only dependent on association with the adaptor proteins DAP10 and DAP12 [1,2,4]. Upon binding to the ligand, signal propagation activates the immunoreceptor tyrosine-based activation motif (ITAM) of DAP12 that recruits and activates Syk [3]. Syk is a relay node phosphorylating and activating several pathways including PI3K/AKT, NF-κB and ERK [5,11,12]. This network also includes JAK-STAT and Wnt/β-catenin pathways, which regulate proliferation and immune responses [13]. Soluble TREM2 (sTREM2) generated by ADAM10/17-mediated proteolytic cleavage of the stalk region terminates TREM2 signaling channels [14]. Membrane-bound TREM2 is responsible for intracellular signaling, but sTREM2 is autocrine or paracrine. For example, it binds to TG2 to induce RhoA deactivation, inhibiting the RhoA-ROCK-GSK3 pathway and reducing tau phosphorylation [15]. sTREM2 is a multifunctional immunomodulator and marker of disease activity, and dynamic fluctuations of sTREM2 levels in CSF and plasma are associated with disease progression [16]. A high CSF sTREM2 correlates with slower cognitive decline and direct neuroprotection by microglia signals [17,18]. A high plasma sTREM2 correlates with liver inflammation, fibrosis, and coronary microvascular disease [19,20]. These findings provide a vital reference for investigating TREM2 in urological pathologies.

Through this network, TREM2 is a “multifunctional regulator” in immune homeostasis. In immune homeostasis, TREM2 suppresses excessive inflammation through inhibition of TNF-α and IL-6 and promotes anti-inflammatory IL-10 expression [21]. In the TME, TREM2 inhibits CD8+ T cell function in an IL-10 dependent fashion by Syk [22]. Phagocytic clearance is another function that facilitates the removal of apoptotic cells and protein aggregates (e.g., amyloid-β) by microglia and macrophages, which helps tissue repair [4,23]. Recently, TREM2 has been recognized as a lipid sensor and metabolic regulator [24]. In AD, TREM2 is required for the transition of microglia to a disease-associated state (DAM), and for the processing of lipids, its absence increases neuroinflammation and damage [10]. Similar metabolic regulation has been demonstrated in obesity and fatty liver disease [25,26]. TREM2-DAP12 signaling supports myeloid cell survival via activation of PI3K/AKT and plays a vital role in physiological processes such as osteoclast differentiation [27].

## 3. The Dual Role of TREM2 in the Tumor Immune Microenvironment: From Functional Paradox to Mechanism Convergence

The function of TREM2 in the tumor immune microenvironment (TME) is highly context dependent, ranging from driving tumor development to triggering tissue protection. In bladder cancer, prostate cancer, and RCC, there exists evidence of convergence among biological phenomena, even among different tumor origins: the tumorigenic function of TREM2 is fundamentally dependent on activation of the PI3K-AKT pathway. The result presents a new framework for broad-spectrum targeted therapies for urological cancer.

### 3.1. TREM2 Function: Dual Cancer-Protection Spectrum

TREM2 is a definitive marker of TAMs. High expression on TAMs contributes to the formation of an immunosuppressive niche, leading to faster tumor progression by inhibiting T cell function and EMT [28,29,30,31]. Clinical studies show that high TREM2 expression correlates with advanced stage, poor prognosis, and resistance to ICIs for melanoma and ovarian, pancreatic, lung, thyroid, and breast cancers [32,33,34,35,36], highlighting its potential as a pan-cancer immunosuppressive target.

However, TREM2 function is not indeterminate but depends on the tissue microenvironment. TREM2 on microglia maintains neural homeostasis and protects neurons by clearing toxic aggregates and fighting inflammation; its loss leads to neurodegeneration [29,37]. In glioblastoma, TREM2 may act protectively by controlling phagocytosis [38,39]. In colorectal cancer, TREM2 may act as a tumor suppressor by inhibiting Wnt/β-catenin signaling [40], while in the liver injury model it may act as an endogenous protective factor by regulating TLR4 inflammation [41]. Non-malignant renal injury (AKI, diabetic nephropathy) also exhibits a protective tendency [42,43,44]. This clear functional plasticity suggests that the ultimate biological output of TREM2 is determined by the micropathological context.

### 3.2. Mechanistic Convergence of Urological Tumors: PI3K/AKT Axis

Compared to other organs, TREM2 has very similar tumorigenic properties in the urological malignancy microenvironment. In bladder cancer, prostate cancer, and RCC, TREM2+ myeloid cells are known to be major drivers of immunosuppression and disease progression [45]. Importantly, the tumorigenic effect is driven by a convergent signaling mechanism: several independent studies suggest that the PI3K-AKT pathway is the central downstream hub for TREM2 in urologic tumors. In prostate cancer:, APOE binds to TREM2 on myeloid cells, forming the APOE-TREM2 axis, which directly activates the PI3K-AKT pathway. This pathway supports tumor cell survival and synergizes with androgen receptor (AR) signaling to generate a powerful immunosuppressive microenvironment [46]. In RCC, TREM2 promotes proliferation and invasion by modulating the PTEN-PI3K/AKT axis [47]. A TME enriched with TREM2+ TAMs correlates closely with T cell suppression and exhaustion. This may explain the poor response of some RCC patients to ICIs [48,49]. In bladder cancer, high expression levels are associated with upregulated PI3K-AKT activity and poor prognosis [50]. In BPH, TREM2-high lipid-enriched macrophages accelerate disease progression by promoting epithelial and stromal proliferation through metabolic and growth signal reprogramming [51], suggesting that the TREM2-PI3K/AKT axis is universally important in urological pathology.

**Figure 1:**
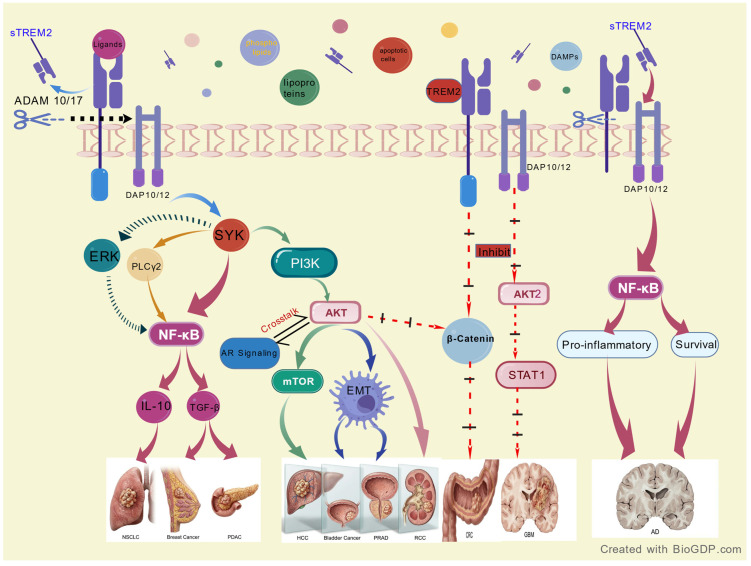
The convergent signaling mechanisms of TREM2 in urological malignancies versus its context-dependent roles in other systems.

This means that the PI3K/AKT pathway may be the “common terminal pathway” (central processor) for TREM2-mediated tumorigenesis in the urological TME. This mechanistic convergence contrasts sharply with the complexity observed in the nervous and digestive systems (Figure 1). This implies that targeting the TREM2-PI3K/AKT pathway may be a “basket” therapy with potential efficacy in multiple urological tumors. Future work should focus on validating this pathway in different preclinical models and linking it with standard treatments (e.g., combination of TREM2 inhibitors with AKT inhibitors, ICIs, or AR targeted agents) in order to improve patient outcomes.

As shown in Figure 1, TREM2 recruits DAP12 and phosphorylates Syk. This leads to a signaling pathway that differs depending on the disease. In urological diseases, this signal preferentially activates the PI3K (p85/p110 subunits)/AKT pathway, which phosphorylates downstream effects like mTOR and GSK-3, driving cell proliferation and survival. In benign cases of BPH, metabolic inputs (lipid loading) are used to steer downstream output toward inflammatory cytokine production rather than uncontrolled proliferation.

## 4. Research Progress of TREM2 in Urological Diseases: From Benign Hyperplasia to Malignant Neoplasms

### 4.1. Bladder Cancer

TREM2 is a known marker of TAMs in bladder cancer and plays a role in shaping the immunosuppressive TME. Early integrative studies show that TREM2 levels in TAMs and some tumor epithelial cells correlate with poor prognosis, EMT, and T cell exhaustion [30]. Further studies suggest that TREM2+ macrophages are highly enhanced and resistant to ICIs in FGFR3-mutated bladder cancer. This is a genotype–immunophenotype link [52]. Functionally, high-resolution myeloid cell atlases show that Trem2 knockout reprograms TME and PD-L1 expression on dendritic cells, supporting TREM2 as a checkpoint [45]. In addition, elevated peripheral blood monocyte TREM2 expression in patients correlates with autonomic imbalance (sympathetic dominance), adding a systemic dimension to tumor-associated immune dysregulation [53]. Together with known mechanisms in other cancers, including the Syk/PI3K/AKT axis [54], TREM2 likely induces tumorigenesis in bladder cancer by activating PI3K/AKT signaling to activate EMT programs, a hypothesis supported by pan-cancer studies [55].

### 4.2. Prostate Cancer

TREM2 is a characteristic of myeloid cells in the prostate cancer immune microenvironment. Early bioinformatics and in vitro studies showed that TREM2 is an independent predictor of poor outcome, suggesting migration and invasion via the PI3K/AKT pathway [56]. Bancaro et al. found that tumor-derived APOE bound TREM2 on myeloid cells to induce “senescent-like neutrophils” with immunosuppressive effects [57]. Wang et al. showed that the APOE-TREM2 axis regulates AR expression via a new DAP12-Src-Syk-STAT3-ROR-γ signaling pathway. This is likely a consequence of the AR-based network [58]. In aggressive subtypes (e.g., cribriform cancer) or metastases, TREM2-rich M2-like macrophages or specific SPP1+/TREM2+ TAM subsets are associated with a poor outcome [46,59]. Genetic models (e.g., MYC-driven and PTEN-loss) confirm that TREM2 signaling may synergize with PTEN loss to induce the hyperactivation of PI3K/AKT, accelerating the progression to castration-resistant prostate cancer (CRPC) [60,61]. Single cells show that TREM2+myeloid cells, C1Q+ TAMs, and senescent-like neutrophils express high levels of IL-10 and TGF-β and suppress CD8+ T cells [57,59]. Co-IP experiments show that TREM2 directly binds AR, enhances AR signaling, and synergizes with PI3K/AKT to shape the immune system [58].

### 4.3. Benign Prostatic Hyperplasia (BPH)

Unlike metabolic functions and immune functions, TREM2 in BPH exemplifies “immunometabolic reprogramming”. Recent single-cell transcriptomic studies show that TREM2 marks a specific subset of lipid-loaded macrophages (foam cells) that accumulate in hyperplastic tissues [51,62]. Instead of being immunologically inert, metabolic foam cells are actively pathogenic. Intracellular lipid accumulation drives the macrophages to secrete pro-inflammatory and pro-fibrotic factors, including TGF-β1, VEGF, and CXCL16, as well as inflammatory cytokines [62]. In fact, these lipid-rich macrophages stimulate the proliferation of prostate epithelial and stromal cells, linking lipid dysregulation to hyperplastic dysregulation [51]. Thus, in BPH, TREM2 is not only a metabolic regulator but also a node linking lipid metabolism to inflammation and tissue overgrowth.

### 4.4. Kidney Diseases

The role of TREM2 in kidney diseases shows extreme “context dependence” (in a benign versus malignant manner) and is clearly protective. Serum sTREM2 is a protective marker [63,64] and inhibits ERK/IL-1 signaling in renal tubular lipid deposition and ferroptosis [42]. In chronic kidney disease (CKD), TREM2+ macrophages can counter-proliferate disease [43], although some contradictory evidence suggests it has a pro-fibrotic role [65]. We hypothesize that in early disease, TREM2+ anti-inflammatory macrophages dominate and play a protective role; in late stages, TREM2 may be highly expressed in fibrotic macrophage subsets contributing to disease. Generally, TREM2+ macrophages in diabetic kidney disease reduce tubular injury via the IL-1β/CD36 direction [42] and may also be modulated by SGLT2 inhibitors [66]. On the other hand, TREM2 is consistently shown to be tumorigenic in RCC. It is highly expressed in tumor cells and TAMs and promotes proliferation and invasion by inhibiting p53 and activating the PTEN-PI3K/AKT axis [47,67]. TREM2+ populations (often co-expressing APOE and C1Q) are highly expressed in high-risk, recurrent, or metastatic clear cell RCC (ccRCC) and have poor survival [49,55,68,69]. Based on these observations, we propose a hypothetical model for the transition from CKD to RCC, where TREM2 expression likely shifts from peritubular macrophages to TAMs, and downstream signaling shifts from protective IL-1β/CD36 to tumorigenic PTEN-PI3K/AKT [42,67].

In summary, TREM2 has a well-defined function in bladder, prostate, and renal cancers: it promotes tumors and IL-suppressors by activating the PI3K/AKT pathway and driving tumor growth and metastasis, according to previous studies [70]. In benign prostate hyperplasia, TREM2 regulates metabolic regulation by lipid metabolism reprogramming and interaction with local inflammation and turns it into a metabolic regulator that drives tissue proliferation. In non-malignant renal diseases, TREM2 protects renal tubular epithelial cell apoptosis and oxidative stress damage, but in chronic kidney disease, it may contribute to renal interstitial fibrosis by activating the transforming growth factor-β pathway, which promotes fibroblast transformation. The bidirectional nature of TREM2 function depends on disease progression and microenvironmental changes (Table 1).

## 5. Therapeutic Potential of TREM2: From Mechanism to Clinical Translation

Since TREM2 shapes the immunosuppressive tumor microenvironment (TME), it is a highly attractive target for tumor immunotherapy. In solid tumors (colorectal cancer [71], gastric cancer [2], hepatocellular carcinoma [72,73,74], ovarian cancer [32]), high expression of TREM2 on tumor-associated macrophages (TAMs) is associated with poor survival and resistance to immunotherapy. Its pro-tumor mechanisms form a multi-level immunosuppression network: intracellularly, TREM2 signaling stabilizes M2-like pro-tumor macrophages by SYK/STAT1 and PI3K/AKT and inhibits their polarization to the anti-tumor M1 phenotype [50,75,76]. Intercellularly, TREM2+ TAMs upregulate PD-L1 on endothelial cells by secreting Galectin-1, blocking recruitment and infiltration of cytotoxic CD8+ T cells to the tumor site [73]. Alternatively, TREM2 promotes the direct differentiation of regulatory T cells, thereby suppressing adaptive immune responses [72]. Recent studies have shown the systemic regulation potential of TREM2, which can indirectly change the gut microbiota (e.g., Ruminococcus) [77]. These multidimensional mechanisms together establish TREM2 as a node for reverse immunosuppression and sensing existing immunotherapies. Interventions targeting TREM2 are rapidly moving from basic research to preclinical and early clinical translation. These include (1) targeting TREM2 directly, where anti-TREM2 monoclonal antibodies or small molecule inhibitors block the TREM2-DAP12 pathway; preclinical studies show that this strategy reprograms TAMs, increases T cell infiltration, and synergizes with ICIs [32,78]; (2) nano-delivery and reprogramming, which involves building nanocarriers targeted with anti-TREM2 antibodies to deliver payloads (e.g., STING agonists and CSF1R inhibitors) to TAMs and to remodel local microenvironment and theranostic monitoring [79,80,81]; and (3) combinatorial cell therapy, which involves engineering CAR-T cells to secrete anti-TREM2 scFv locally, thus neutralizing myeloid immunosuppression while targeting tumor cells [82]. Currently, the most advanced clinical agent is the monoclonal antibody PY314. Its Phase Ib combination with pembrolizumab for advanced RCC has shown preliminary safety [48]. However, monotherapy efficacy is limited, and combination therapy is needed.

## 6. Discussion and Future Perspectives

### 6.1. Synthesis of Mechanistic Convergence

The evidence presented here indicates a “mechanistic convergence” of TREM2 signaling in urological malignancies. Unlike neurodegenerative diseases or other solid tumors, where TREM2 function is highly context-dependent and plastic, TREM2 is consistently a pro-tumorigenic driver in bladder, prostate, and renal cancer [30,46,47,56]. We show that this effect propagates through the PI3K/AKT hub, encouraging EMT and immunosuppression [54,55]. This consistency distinguishes urological malignancies from benign conditions like BPH (where TREM2 is primarily metabolic) and AKI (where it acts as a protective factor) [51,63].

In addition, other pathways, such as NF-κB and Wnt/β-catenin, play a role in TREM2 signaling in a variety of systems; however, current data show that in the urological tract, these pathways frequently converge with or act in parallel with the PI3K/AKT pathway to promote malignant behavior [5,58]. The strength of evidence depends on cancer types; for example, the dominant role of PI3K/AKT is supported by direct functional validation, such as specific inhibitors and knockdown assays in prostate cancer [56]. In bladder and renal cell carcinomas, the evidence comes mostly from robust transcriptomic analyses and pathway enrichment studies that consistently show PI3K/AKT is the top dysregulated signature associated with TREM2. This convergence provides a good reason for developing “basket” therapeutics targeting the TREM2-PI3K/AKT axis in different types of cancers, possibly overcoming the heterogeneity seen in individual tumor types.

### 6.2. Renal Safety Considerations in TREM2-Targeted Therapy

Safety remains a primary concern due to the opposing roles of TREM2 in RCC (where it is tumorigenic) and kidney injury (where it is protective). Many patients with RCC undergo partial or radical nephrectomy, placing them at an increased risk for acute kidney injury (AKI) or chronic kidney disease (CKD) in the remaining kidney. Systemic inhibition of TREM2, such as through the use of anti-TREM2 antibodies like PY314, could inadvertently obstruct the protective anti-inflammatory and repair functions of TREM2 in healthy renal tissue, potentially worsening renal injury [42,48]. Therefore, future clinical trials must rigorously monitor markers of renal function. Additionally, the development of tumor-targeted delivery systems, including nanotherapeutics or antibody-drug conjugates (ADCs), may be essential for precisely targeting tumor-associated macrophages while preserving the protective TREM2 populations in healthy renal tissue [83,84].

### 6.3. Determinants of TREM2 Functional Switching: A Context-Dependent Framework

The functional heterogeneity of TREM2 (protective of tissue injury, pathogenic driver of cancer) depends on microenvironmental factors. First, ligand availability is critical; TREM2 activates in response to anionic lipids (e.g., phosphatidylserine on apoptotic cells) and lipoproteins (e.g., APOE-LDL complexes). In the tumor microenvironment, the dysregulated abundance of specific lipids likely sustains pathogenic signaling, as seen in lipid-loaded macrophages in hyperplastic prostates [51]. Second, co-factor dependence dictates downstream outputs; while canonical TREM2 signaling relies on DAP12 coupling to activate SYK and PI3K/AKT [56], alternative associations or DAP12-dependent pathways may emerge under stress. Third, local cytokine profiles (e.g., TGF-β, IL-4 vs. IFN-γ) shape the transcriptional landscape of TREM2+ macrophages (e.g., TGF-rich environments can drive these cells towards a pro-fibrotic and immunosuppressive phenotype [62]). Future work should focus on mapping these “molecular switches” to establish a precise therapeutic window.

### 6.4. Future Directions and Challenges

To target TREM2 as a therapeutic candidate, however, one of the biggest challenges lies in its context-dependent function. Treatment strategies must be tailored to the specific disease contexts. The most representative contrast is between cancer and Alzheimer’s disease: in most solid tumors, TREM2 is an immunosuppressor that needs to be attacked; in Alzheimer’s disease, TREM2 acts as a critical receptor maintaining microglial function and clearing toxic proteins, using agonists developed for neuroprotection [85,86,87]. Even in tumors, its role can vary—for instance, in glioblastoma, some studies show that TREM2 regulates phagocytic function rather than directly mediating immunosuppression [39]. A systematic review analyzes TREM2’s dual role in both diseases, highlighting the concept of disease-based precision regulation [88]. Future development steps include the following: (1) single-cell and spatial omics techniques to analyze the functional status of TREM2+ cells at the patient level; (2) the development of tumor-responsive (e.g., pH/enzyme activation) or tissue-specific targeted drugs to maximize the window of therapeutic effect; (3) early clinical trials (e.g., limited success of PY314 combined with pembrolizumab in renal cancer), which suggest that single-targeting TREM2 may not suffice in an established strong immunosuppressive microenvironment, as tumors may develop resistance by compensatory mechanisms [48]; and (4) mechanism-based exploration of more effective combination therapies. For example, in prostate cancer, this may involve combined targeting of TREM2 and AR signaling; in renal or bladder cancer, this may involve combining inhibitors with anti-angiogenic drugs, other immunomodulators (e.g., STING agonists), or chemotherapy, thus optimizing treatment sequence [89].

In the urinary system (in bladder cancer and nephropathy), urine acts as a natural window for diagnostic tests. Detecting urinary sTREM2 could be a noninvasive method for early bladder cancer detection, recurrence monitoring, or AKI warning [63,64]. There is no standard for TREM2 activity detection or clinical cutoff values, which makes it difficult to screen beneficiaries. A standard detection system for TREM2 expression (tissue/liquid biopsy) should be established through multicenter cohort studies. By leveraging artificial intelligence (AI) and machine learning models to integrate genomic data (e.g., mutations in FGFR3 immune microenvironment characteristics (e.g., CD8+ T cell infiltration)), downstream pathway activity can be used to construct predictions for clinical trial patient enrollment and treatment strategy selection [90,91,92]. TREM2 protects benign conditions such as acute renal injury, which conflicts with its oncogenic role in renal cell carcinoma, and precise differentiation of the pathology of therapeutic strategies is needed. Systematic studies on the dynamic functional switching mechanisms of TREM2 in “benign hyperplasia-malignant mechanistic parallels” (BPH to prostate cancer, CKD to renal cell carcinoma) are needed to identify the key molecular switches from protective to pathogenic effects and pinpoint precise spatiotemporal windows for safe interventions.

## 7. Conclusions

In summary, this review elucidates the pivotal role of TREM2 as a “double-edged sword” within the urological system. We identify a critical functional dichotomy: in malignancies, including bladder, prostate, and kidney cancers, TREM2 consistently acts as an oncogenic driver. This effect is mediated through a convergent PI3K/AKT signaling hub, which orchestrates epithelial–mesenchymal transition (EMT), reinforces immunosuppression, and promotes therapy resistance. Conversely, in benign conditions, TREM2 functions as an immunometabolic regulator that drives stromal proliferation in benign prostatic hyperplasia (BPH) and serves as a protective factor that facilitates tissue repair in renal injury.

Current research is moving quickly from mechanistic exploration to clinical translation. Using single-cell sequencing and spatial omics, future work can explore the heterogeneity of TREM2+ subpopulations and provide precise patient stratification. Clinically, “mechanistic convergence” validates TREM2 as a promising broad-spectrum target and noninvasive urinary biomarker. The ultimate success of targeting TREM2 lies in a combination of rational therapies (anti-TREM2 antibodies with immune checkpoint inhibitors (ICIs) or targeted agents), which can overcome complex immune evasion in urological tumors and translate this promising target into practical survival benefits.

## Figures and Tables

**Table 1:** The functional landscape, mechanistic convergence, and therapeutic potential of TREM2 in urological diseases versus other cancers.

Disease Type	Role	Cell Source	Key Signaling Mechanism	Pathological Outcome	Potential Combination Strategy	Ref.
I. Urological System						
Prostate Cancer	Pro-tumorigenic	TAMs; PMN-MDSCs	APOE-TREM2-AR Axis; PI3K/AKT; STAT3-RORγ	Enhances AR signaling; Promotes immunosuppression	Anti-TREM2 + AR Antagonists	[56,57,58]
Bladder Cancer	Pro-tumorigenic	TAMs	PI3K/AKT → EMT; FGFR3 mutation link	Drives EMT; Induces T cell exhaustion and ICB resistance	Anti-TREM2 + Anti-PD-1/L1	[30,45,52]
Renal Cell Carcinoma	Pro-tumorigenic	TAMs (C1Q+), Tumor cells	PTEN-PI3K/AKT; p53 inhibition	Promotes proliferation; Poor prognosis in ccRCC	Anti-TREM2 + AKT Inhibitors	[47,68,69]
BPH (Benign)	Pathogenic	Lipid-loaded Macrophages	Lipid Metabolism; Growth factor signaling	Promotes stromal and epithelial hyperplasia (distinct from malignancy)	Metabolic Modulators + 5α-RIs	[51,62]
AKI/CKD	Protective	Tubular Macrophages	Inhibition (⊣) of ERK/IL-1β; CD36 downregulation	Protects against ferroptosis; Promotes tissue repair	TREM2 Agonists	[7,42,64]
II. Other Cancers						
Glioblastoma	Protective	Microglia	Sphingolipids → SYK → AKT2	Drives M1-like polarization; Enhances phagocytosis	TREM2 Agonists	[39]
Colorectal Cancer	Protective	TAMs	Inhibition (⊣) of Wnt/β-Catenin	Inhibits tumor cell proliferation	TREM2 Agonists	[40]
NSCLC/Breast Cancer	Pro-tumorigenic	TAMs	NF-κB → IL-10/TGF-β	Classical immunosuppressive TME	Anti-TREM2 + ICIs/Chemo	[22,55]

Abbreviations: AR, androgen receptor; TAMs, tumor-associated macrophages; PMN-MDSCs, polymorphonuclear myeloid-derived suppressor cells; EMT, epithelial–mesenchymal transition; ICB, immune checkpoint blockade; ccRCC, clear cell renal cell carcinoma; 5α-RIs, 5α-reductase inhibitors; NSCLC, non-small cell lung cancer; TME, tumor microenvironment. Symbols: → indicates activation or downstream induction; ⊣ indicates inhibition.

## Data Availability

No new data were created or analyzed in this study. Data sharing is not applicable to this article.
